# Ultrasonographic evaluation of chronic shoulder pain after breast cancer surgery: single center, cross-sectional study

**DOI:** 10.1038/s41598-020-73769-8

**Published:** 2020-10-08

**Authors:** Jung Hun Kim, Se Hee Kim, Hae-Rim Kim, Sang-Heon Lee, So Young Yoon, Jung-Hyun Yang, Young Bum Yoo, Kyoung Sik Park, Sang Eun Nam, Semie Hong, Hong Ki Min

**Affiliations:** 1 Department of Internal Medicine, Konkuk University Medical Center, Seoul, 05030 Republic of Korea; 2 Division of Rheumatology, Department of Internal Medicine, Konkuk University Medical Center, 120-1 Neungdong-ro (Hwayang-dong), Gwangjin-gu, Seoul, 05030 Republic of Korea; 3 Division of Rheumatology, Department of Internal Medicine, Research Institute of Medical Science, Konkuk University School of Medicine, Seoul, 05030 Republic of Korea; 4 Division of Oncology, Department of Internal Medicine, Research Institute of Medical Science, Konkuk University School of Medicine, Seoul, 05030 Republic of Korea; 5 Department of Surgery, Konkuk University School of Medicine, Seoul, 05030 Republic of Korea; 6 Department of Radiation Oncology, Konkuk University Medical Center, Seoul, 05030 Republic of Korea

**Keywords:** Oncology, Rheumatology

## Abstract

Chronic shoulder pain is a common complication in breast cancer patients after surgery. Chronic shoulder pain after breast cancer surgery was formerly considered as neuropathic pain, however the pathophysiology including structural damages has not been assessed comprehensively. We hypothesized that the structural change could be one of the cause of shoulder pain after breast cancer surgery and evaluated various ultrasonography findings of the shoulder in breast cancer patients with chronic shoulder pain. Patients who were suffering from chronic shoulder pain on unilateral side for at least 3 months after breast cancer surgery were enrolled from a single tertiary hospital. Demographic and clinical data were collected at the baseline. Articular and adjacent structures of both shoulders (painful and contralateral side) were evaluated by ultrasonography. The ultrasonography findings were compared between painful and contralateral sides. Logistic regression analysis was performed to determine the factors associated with abnormal ultrasonography findings. Fifty-two female patients (average age of 55) were enrolled. Significantly more abnormal ultrasonography findings were observed in the painful side than in the contralateral side [39 (75.0%) vs 11 (21.2%), *P* < 0.001]. The coracohumeral ligament was significantly thicker in the painful side than in the contralateral side (2.48 ± 0.69 vs 1.54 ± 1.25 mm, *P* < 0.001); adhesive capsulitis was also more frequent in the painful side [14 (26.9%) vs 0, *P* < 0.001]. Furthermore, patients with a history of breast cancer surgery on the ipsilateral side were associated with abnormal ultrasonography findings and adhesive capsulitis. This study is the first to evaluate ultrasonography in patients with chronic shoulder pain after breast cancer surgery. The results showed that ultrasonography could reveal several structural problems in these patients.

## Introduction

Breast cancer is the second most commonly diagnosed malignancy, and the most common cause of death related to malignancy among females in Korea^1^. These trends are similar worldwide^2^. The treatment modalities of breast cancer include surgery, radiotherapy, chemotherapy, and hormone therapy. Although remarkable developments have been achieved in adjuvant therapy of breast cancer, surgery is still important to manage breast cancer^3^. Breast cancer surgery can be divided into curative and palliative, as well as into mastectomy and breast conserving surgery (BCS). Chronic shoulder pain after breast cancer surgery was usually termed as post mastectomy pain syndrome (PMPS). PMPS was a relatively common complication in breast cancer patients who underwent surgery and affects about 25–60% of patients^4^. According to the International Association for the Study of Pain (IASP), chronic shoulder pain after breast cancer surgery can be defined as chronic pain lasting at least 3 months after the surgery^5^. It frequently involves the axilla, shoulder, arm, and chest wall in the ipsilateral side of the breast surgery^6^. Recently, IASP classified chronic pain into seven subcategories, and chronic shoulder pain after breast cancer surgery was defined as “chronic pain” in the 11th edition of International Classification of Diseases (ICD-11)^5^. Patients with chronic shoulder pain after breast cancer surgery have a poor quality of life (QoL) and require more medication^7^.

Chronic shoulder pain after breast cancer surgery is a complex chronic pain, and it is generally thought as a neuropathic pain that is localized to the breast and nearby structures^8^. The pathophysiology of chronic shoulder pain after breast cancer surgery is complex, and several factors contributing to it have been studied before. Studies from Denmark showed that young age (under 40), adjuvant radiotherapy, and axillary lymph node (LN) dissection were associated with chronic shoulder pain after breast cancer surgery^9^. Another study reported that chronic pain history and young age were significantly associated with chronic shoulder pain after breast cancer surgery^10^. Nerve damage during surgery, fibrosis during healing, and radiotherapy were suggested as the underlying reasons of chronic shoulder pain after breast cancer surgery^11,12^. Several medications commonly used for neuropathic pain such as antidepressants and anti-epileptics have been used to treat chronic shoulder pain after breast cancer surgery^8^; however, there are still some patients who do not respond to these medications. Therefore, evaluating the structural change by imaging modalities in patients with chronic shoulder pain after breast cancer surgery is important to understand its pathophysiology and develop novel therapeutic modalities.

Physicians can consider several structural changes as cause of shoulder pain after breast cancer surgery. Patients often experience decreased strength and range of motion of the shoulder after breast cancer surgery^13^. Adhesive capsulitis is often known as “frozen shoulder,” because it has the characteristics of progressive pain and limited range of motion^14^. One study revealed that approximately 10% of breast cancer patients suffered from adhesive capsulitis, and a history of mastectomy was a major factor associated with adhesive capsulitis^15^. However, previous study diagnosed adhesive capsulitis only by a medical chart review and did not confirm adhesive capsulitis by imaging analysis^15^. Previous study suggested that shoulder pain could be arise from structural change after breast cancer surgery.

Usually, imaging modalities are used to access pain in periarticular structures. There are many modalities to evaluate pathologic change in the shoulder, including adhesive capsulitis, and the most commonly used modalities are magnetic resonance imaging (MRI), magnetic resonance (MR) arthrography, and ultrasonography^14,16,17^. The thickness of the coracohumeral ligament is useful in evaluating adhesive capsulitis and can be easily measured by ultrasonography^16,18^. Ultrasonography is a non-invasive and useful imaging modality that can produce both static and dynamic images. Musculoskeletal ultrasonography of the shoulder can reveal structural changes in surrounding tendons, bursa, and joints^19^. Ultrasonography has more advantages than computed tomography (CT) and MRI in several aspects: (1) it can provide real-time visualization and enable immediate treatment via ultrasonography guided injections, (2) it is free from radiation exposure, (3) and it is relatively cheaper than CT and MRI^19^.

We hypothesized that chronic shoulder pain after breast cancer surgery is not always the result of neuropathic pain, but could arise from structural changes in the shoulder. To prove our hypothesis, we evaluated the ultrasonography of the shoulders of breast cancer patients with chronic shoulder pain after breast surgery. We focused on structural changes in joints and structures adjacent to the shoulder in these patients and compared them with the contralateral shoulder of the same patients.

## Materials and methods

### Patients

Breast cancer patients were recruited from single tertiary university hospital, Konkuk University Medical Centre, from November 2019 to February 2020. Inclusion criteria were as follows: (1) pathologic confirmation of breast cancer, (2) unilateral upper arm to shoulder pain after breast cancer surgery, (3) pain lasting for at least 3 months, (4) age over 18, and (5) unilateral breast cancer. Patients who had definite cause of pain such as lymphedema, breast cancer recurrence, or arthritis were excluded. All participants provided written informed consent prior to enrolment. The study was conducted in accordance with the Declaration of Helsinki (1964) and was approved by the Institutional Review Board of the Konkuk University Medical Center (IRB number: 2019-09-037).

### Physical examinations and medical chart review

Medical history of cancer stage, surgery type, extent of LN dissection, chemotherapy, radiotherapy, and hormone therapy were checked by reviewing electronic medical records. A questionnaire was provided to each patient to determine the baseline demographic characteristics, pain site, pain duration, numerical rating scale (NRS) for pain intensity, and dominant hand. Physical examinations of the shoulder including Speed’s test (positive for biceps brachii tendon instability or tendinitis), Lift-off test (positive for subscapularis tear or tendinopathy), Jobe’s test (positive for supraspinatus tear or tendinopathy), Drop arm sign test (positive for infraspinatus tear or tendinopathy), and Neer’s test (positive for subacromial impingement syndrome) were performed. The exact procedures for the physical examinations are described elsewhere^20^.

### Musculoskeletal ultrasonography

Trained rheumatologist, Min, examined all the musculoskeletal ultrasonography images of the patients. Articular joints and the adjacent enthesis and bursa of the shoulder were accessed via ultrasonography, bilaterally on each patient. Tenosynovitis, partial tear or tendinosis of the rotator cuff, subacromial-subdeltoid bursitis, synovitis or effusion on the glenohumeral and acromioclavicular joint, and the thickness of the coracohumeral ligament were visualized and checked according to the ultrasound technique guideline^21^. Adhesive capsulitis was defined when the coracohumeral ligament thickness exceeded 3 mm^16,18^, and limited range of motion for external and internal rotation, forward flexion, and abduction was confirmed on physical examination. A multifrequency linear array transducer (8–13 MHz) of Logiq E (Philips Healthcare, Amsterdam, Netherlands) was used in the B mode or power doppler mode.

### Statistical analysis

Continuous variables (age, duration, BMI, and pain NRS) were compared by Student’s T test or the Mann–Whitney test and displayed as mean ± standard deviation. Categorical variables such as gender, painful shoulder side, cancer stage, operation side, proportion of breast surgery type/reoperation/lymph node dissection/radiation therapy/chemotherapy/hormone therapy/dominant hand were compared using the chi-square test or Fisher’s exact test. Logistic regression analysis was performed to determine the factors associated with abnormal ultrasonography findings. *P* values under 0.05 were considered statistically significant, and all analyses were performed by SPSS statistics version 20 (SPSS, Chicago, Illinois, USA).

## Results

### Baseline demographics and characteristics associated with breast cancer

**Figure 1:**
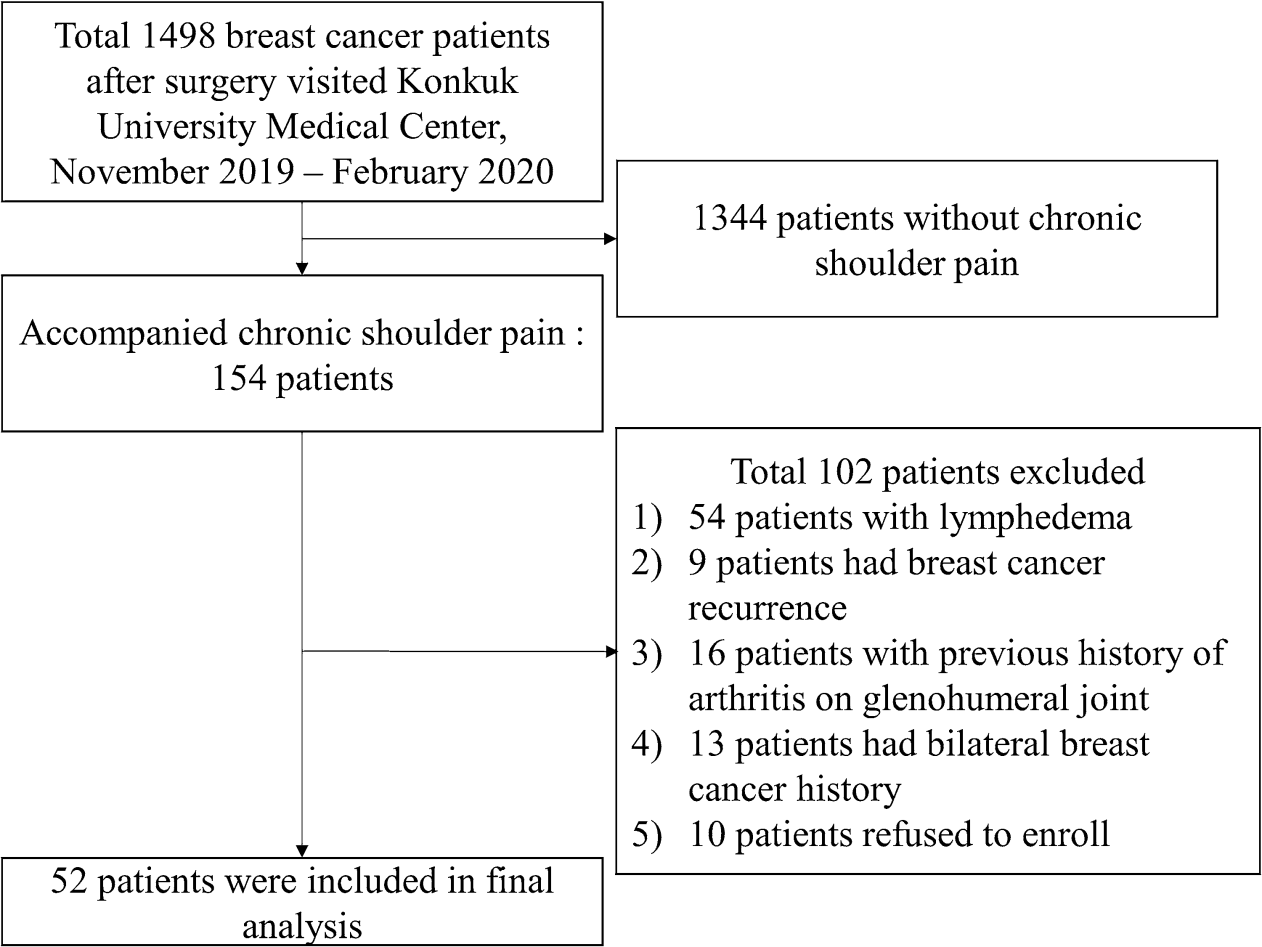
Flow chart for inclusion and exclusion of breast cancer patients.

Among 1498 patients, 154 breast cancer patients had chronic shoulder pain. After excluding 102 patients, a total of 52 female patients were included in the final analysis (Fig. 1). They experienced pain for an average duration of 1.7 years, and most of the patients complained about pain on the same side as the breast cancer surgery site (88.5%). 43 patients had received radiotherapy. Other characteristics are presented in Table 1.

**Table 1:** Baseline characteristics of patients with chronic shoulder pain after breast cancer surgery.

	Total breast cancer patients (N = 52)
Age (years)	54.9 ± 9.1
Female gender (N, %)	52 (100%)
Duration since breast cancer surgery (years)	4.4 ± 4.7
Duration of shoulder pain (years)	1.7 ± 2.4
BMI (kg/m^2^)	23.8 ± 3.6
Pain on ipsilateral to cancer surgery side	46 (88.5%)
Pain NRS (0–10)	4.2 ± 2.0
**Cancer stage**	
Unknown	2
Stage 0	2
Stage 1	10
Stage 2	24
Stage 3	13
Stage 4	1
Operation side (right side, %)	30 (58.8%)
BCS (N, %)	36 (69.2%)
Reoperation (N, %)	7 (13.5%)
**Lymph node dissection (N, %)**	49 (94.2%)
Sentinel lymph node dissection	14
Extensive lymph node dissection	35
Radiation therapy (N, %)	43 (82.7%)
Neoadjuvant chemotherapy (N, %)^a^	8 (15.4%)
Adjuvant chemotherapy (N, %)^a^	35 (67.3%)
Hormone therapy (N, %)	37 (71.2%)
Dominant hand (Right side, %)	46 (88.5%)

Continuous variables were presented as mean ± standard deviation.

*BCS* breast conserving surgery, *BMI* body mass index.

^a^Four patients had history of both adjuvant and neoadjuvant chemotherapy.

### Physical examination of shoulder

All the enrolled patients underwent routine physical examination before ultrasonography. The results of their physical examination are shown in Table 2. Patients were most frequently positive for the Speed’s test followed by Neer’s test and Jobe’s test.

**Table 2:** Physical examination of patients with chronic shoulder pain after breast cancer surgery.

	Total breast cancer patients (N = 52)
Speed's test	16 (30.8%)
Lift-off test	10 (19.2%)
Jobe's test	14 (26.9%)
Drop arm test	8 (15.4%)
Neer's test	15 (28.8%)

### Musculoskeletal ultrasonography findings

**Figure 2:**
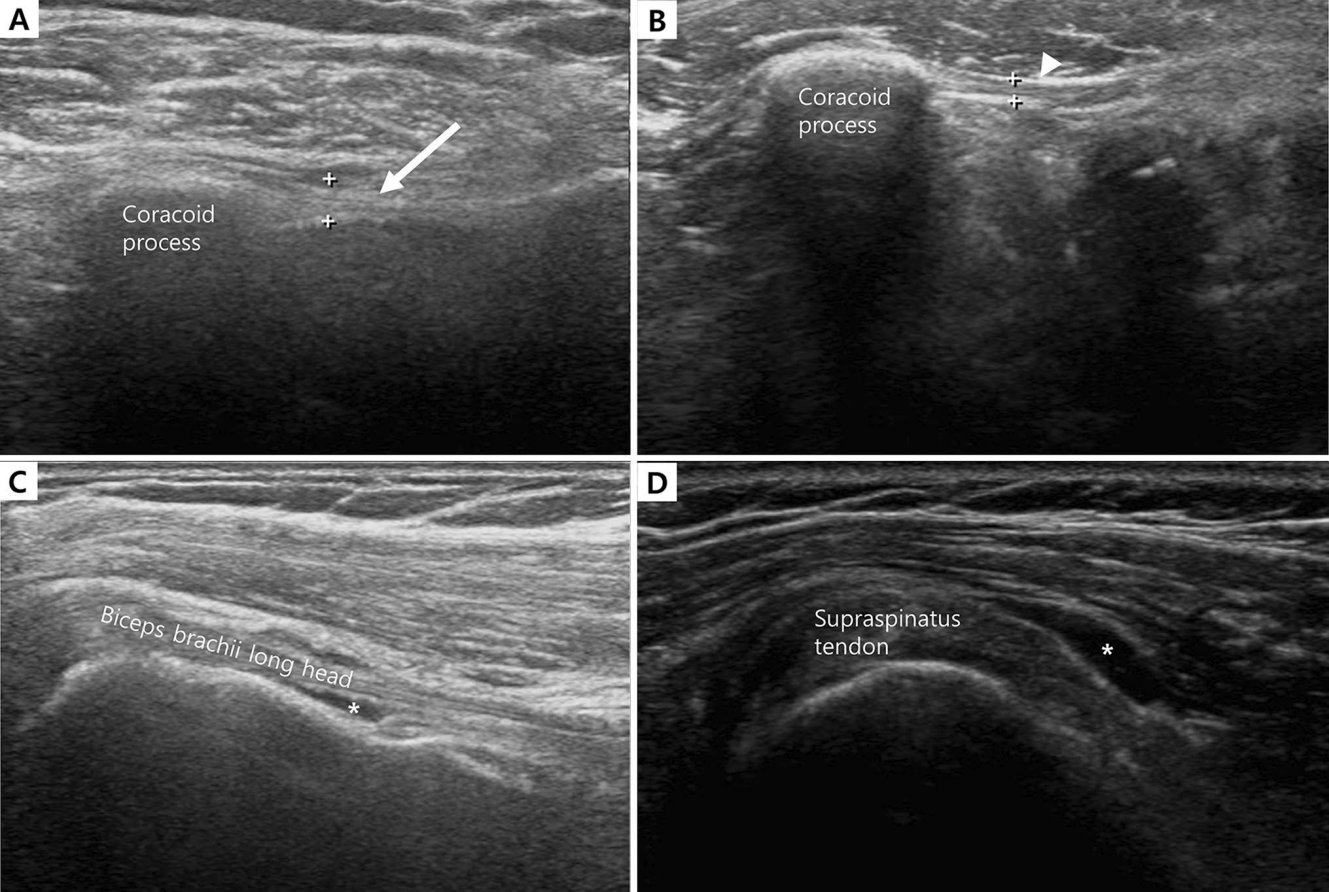
Representative image of shoulder ultrasonography in patients with chronic shoulder pain after breast cancer surgery. (**A**) Increased thickness of coracohumeral ligament (white arrow) in painful shoulder. (**B**) Normal thickness in contralateral side. (**C**) Peritendinous effusion (asterix) indicating biceps tenosynovitis. (**D**) Fluid collection (asterix) at subacromial-subdeltoid (SASD) bursa.

Table 3 compares the ultrasonography findings between the painful shoulder and the contralateral shoulder. Significantly more abnormal ultrasonography findings were observed in the painful side than in the contralateral side [39 (75.0%) vs 11 (21.2%), *P* < 0.001]. Biceps tenosynovitis and supraspinatus tendon were the most frequently seen pathologies in ultrasonography. Infraspinatus partial tear and glenohumeral joint synovitis were rarely observed. These tendencies were also observed in physical examinations. The coracohumeral ligament was significantly thicker in the painful side than in the contralateral side (2.48 ± 0.69 vs 1.54 ± 1.25 mm, *P* < 0.001), and adhesive capsulitis was more frequent in the painful side [14 (26.9%) vs 0, *P* < 0.001]. The correlation between physical examination and ultrasonography was non-significant (R = 0.55, *P* = 0.26 for Speed’s test vs biceps ultrasonography; R = 0.43, *P* = 0.35 for Lift-off test vs subscapularis ultrasonography; R = 0.39, *P* = 0.54 for Jobe’s test vs supraspinatus ultrasonography; R = 0.42, *P* = 0.35 for Drop arm sign test vs infraspinatus ultrasonography). Figure 2 shows the representative images of ultrasonography findings in breast cancer patients with chronic shoulder pain.

**Table 3:** Comparison of ultrasonography findings between painful shoulder and contralateral shoulder.

	Painful shoulder	Contralateral shoulder	*P*
Biceps tenosynovitis	24 (46.2%)	5 (9.6%)	< 0.001
Subscapularis tendinosis	6 (11.5%)	1 (1.9%)	0.112
Supraspinatus partial tear	24 (46.2%)	7 (13.5%)	< 0.001
Infraspinatus partial tear	2 (3.8%)	0	0.495
SASD bursitis	9 (17.3%)	4 (7.7%)	0.138
Glenohumeral joint synovitis	2 (3.8%)	0	0.495
Acromio-clavicular joint effusion	1 (1.9%)	0	1.000
Coracohumeral ligament thickness (mm)	2.48 ± 0.69	1.54 ± 1.25	< 0.001
Adhesive capsulitis	14 (26.9%)	0	< 0.001
Abnormal ultrasonography findings	39 (75.0%)	11 (21.2%)	< 0.001

Continuous variables were presented as mean ± standard deviation.

*SASD* subacromial-subdeltoid.

### Factors associated with abnormal ultrasonography finding and adhesive capsulitis

In the enrolled patients, a history of breast cancer surgery on the ipsilateral side of the painful shoulder was associated with adhesive capsulitis (OR 5.76, *P* = 0.01, Table 4), and abnormal ultrasonography findings (OR 9.12, *P* < 0.01, Table 5). However, other factors such as radiotherapy, chemotherapy, cancer stage, degree of LN dissection, and dominant hand did not show significant association.

**Table 4:** Associated factors with adhesive capsulitis in patients with chronic shoulder pain after breast cancer surgery.

	Odd ratio	95% CI	*P*
Age	0.99	0.93, 1.06	0.82
BMI	1.02	0.86, 1.21	0.83
Breast cancer surgery on ipsilateral side of painful shoulder	5.76	1.50, 22.12	0.01
Surgery type (BCS vs total mastectomy)	0.55	0.13, 2.33	0.41
Duration from surgery to first symptom (> 5 years)	0.24	0.05, 1.25	0.09
LN dissection level (sentinel LN biopsy vs 1/2/3 level)	1.46	0.37, 5.75	0.59
Radiation therapy	3.03	0.34, 27.21	0.32
Neoadjuvant chemotherapy	1.69	0.35, 8.28	0.52
Adjuvant chemotherapy	0.76	0.21, 2.80	0.68
**Cancer stage**			
Stage 0/1 (reference)			
Stage 2	1.00	0.23, 4.35	1.00
Stage 3/4	0.33	0.05, 2.27	0.26

*BCS* breast conserving surgery, *BMI* body mass index, *CI* confidence interval, *LN* lymph node.

**Table 5:** Associated factors with abnormal findings of shoulder ultrasonography in patients with chronic shoulder pain after breast cancer surgery.

	Odd ratio	95% CI	*P*
Age	1.06	0.97, 1.15	0.19
BMI	0.85	0.71, 1.02	0.08
Breast cancer surgery on ipsilateral side of painful shoulder	9.12	3.72, 22.37	< 0.01
Surgery type (BCS vs total mastectomy)	0.95	0.24, 3.76	0.94
Duration from surgery to first symptom (> 5 years)	1.17	0.30, 4.54	0.82
LN dissection level (sentinel LN biopsy vs 1/2/3 level)	1.6	0.40, 6.33	0.51
Radiation therapy	1.98	0.40, 9.77	0.4
Neoadjuvant chemotherapy	0.52	0.11, 2.57	0.42
Adjuvant chemotherapy	1.53	0.41, 5.74	0.53
**Cancer stage**	0.38	0.09, 1.66	0.20
Stage 0/1 (reference)			
Stage 2	3.57	0.74, 17.19	0.11
Stage 3/4	1.79	0.35, 9.13	0.49

*BCS* breast conserving surgery, *BMI* body mass index, *CI* confidence interval, *LN* lymph node.

## Discussion

In the present study, we investigated the structural abnormalities or damages in patients with chronic shoulder pain after breast cancer surgery, for the first time. Biceps tenosynovitis, supraspinatus partial tear, and adhesive capsulitis were the top three common abnormal ultrasonography findings in these patients. We revealed the structural damages present in the shoulder of these patients and confirmed our findings using ultrasonography. These findings may provide a new perspective to understand the etiology of chronic shoulder pain after breast cancer surgery and to develop novel therapeutic approaches for it.

Chronic pain syndrome has attracted considerable attention recently; it is perceived as a unique disease entity that should be treated promptly. IASP defines “chronic pain” as a pain lasting for at least 3 months, and chronic shoulder pain after breast cancer surgery can be categorized into “chronic postsurgical or posttraumatic pain” according to ICD-11^5^. Managing chronic shoulder pain after breast cancer surgery is very important, because this is associated not only with poor QoL and a high cost on medication, but also with a low employment rate^7,22^. Therefore, understanding the pathophysiology of chronic shoulder pain after breast cancer surgery and prompt treatment are crucial; however, the etiology of chronic shoulder pain after breast cancer surgery has not yet been fully revealed. About half of the patients with chronic pain after breast cancer surgery showed neuropathic features such as paresthesia, allodynia, and phantom sensation^7^; therefore, chronic shoulder pain after breast cancer surgery was assumed as neuropathic pain. However, a recent study showed adhesive capsulitis occurred in about 10% of patients who underwent breast cancer surgery^15^. Although the aforementioned study showed the possibility of structural damage in breast cancer patients with chronic pain, the diagnosis of adhesive capsulitis was based only on medical charts 15. In the present study, for the first time, we visualized and confirmed the existence of several structural problems in the shoulder of breast cancer patients after surgery. Therefore, chronic shoulder pain in patients after breast cancer surgery should not always be concluded as neuropathic pain; the use of imaging studies such as ultrasonography can discriminate cause of shoulder pain in these patients.

Pharmacological and surgical approaches have been used to treat chronic shoulder pain after breast cancer surgery. Even though several medications targeting the neuropathic feature of chronic shoulder pain after breast cancer surgery as well as autologous fat grafting to reduce scar retraction have been attempted, some of the patients do not respond to these treatment modalities. The standard treatment strategy for chronic shoulder pain after breast cancer surgery has not yet been established^8^. Musculoskeletal ultrasonography has advantages over other imaging modalities, as it can provide real-time visualization of pathologic structures and guide target tissues for glucocorticoid injection^19^. In adhesive capsulitis treatment, intra-articular glucocorticoid injections showed superior therapeutic effect than NSAIDs or physiotherapy in improving shoulder function^23,24^. In biceps tenosynovitis, ultrasonography guided glucocorticoid injection showed higher efficacy than blind injection^25^. Partial tear of the rotator cuff can be treated via several modalities, such as ultrasonography guided glucocorticoid or plasma injection, oral NSAIDs, or extracorporeal shockwave therapy^26–29^. Our findings suggest that proper therapy should be chosen according to the cause of pain in patients with chronic shoulder pain after breast cancer surgery.

Previous studies have demonstrated several risk factors linked with chronic shoulder pain after breast cancer surgery. Young age, radiotherapy, choosing mastectomy over BCS, history of chronic pain, and axillary LN dissection were associated with an increased risk of chronic shoulder pain after breast cancer surgery^7,9,10,22^. In the present study, abnormal ultrasonography findings were only significantly associated with the ipsilateral side of breast cancer surgery. None of the other considered factors such as, radiotherapy, LN dissection over sentinel nodes, and surgery type showed significant association. This may be because of the small sample size and the similarity in the treatment modalities for breast cancer in the patients enrolled. About 80% of the enrolled patients had undergone radiotherapy/chemotherapy (adjuvant or neoadjuvant). Further studies with a larger and more diverse sample may clarify the above discussed inconsistencies.

Chronic shoulder pain after breast cancer surgery was usually assumed as a pain on the ipsilateral side of the breast cancer surgery site. Interestingly, in the present study, about 10% of patients suffered shoulder pain on the contralateral side of the breast cancer surgery site. This may indicate that the shoulder pain in patients with breast cancer is not always related with cancer surgery. Another hypothesis is that patients may unconsciously overprotect the ipsilateral side shoulder of breast cancer surgery and overuse the contralateral side shoulder. Further study estimating mechanical loading on ipsilateral and contralateral side shoulder after breast cancer surgery may give novel insight^30^. Additionally, ultrasonography should be performed on painful and contralateral sides simultaneously. Comparing ultrasonography of both shoulders helps to identify structural damages in the painful shoulder.

The ultrasonography finding and physical examination did not correlate in the present study. These may arise from relatively low accuracy of physical examination than ultrasonography when detecting structural pathologies of shoulder^31^. Currently, the better diagnostic power of ultrasonography is well known and ultrasonography can guide toward the exact site for injection. Therefore, ultrasonography should be actively used in patients with shoulder pain.

This study has several limitations. First, the number of enrolled patients were relatively small. Second, the study was conducted in a cross-sectional manner and follow-up data were not available. Attempting a therapeutic approach via an ultrasonography guided injection and observing the follow-up results may provide novel insights for treating chronic shoulder pain after breast cancer surgery in the future.

This study evaluated the ultrasonography of the shoulder in patients with chronic shoulder pain after breast cancer surgery for the first time. In patients with chronic shoulder pain after breast cancer surgery, several structural damages were observed via ultrasonography in the shoulder of the painful side. Therefore, physicians should not conclude chronic shoulder pain as neuropathic pain in breast cancer patients after surgery but should also consider structural damage. In terms of evaluating structural damage, musculoskeletal ultrasonography may be a useful screening tool for these patients.
